# CT-based data generation for foreign object detection on a single X-ray projection

**DOI:** 10.1038/s41598-023-29079-w

**Published:** 2023-02-02

**Authors:** Vladyslav Andriiashen, Robert van Liere, Tristan van Leeuwen, K. Joost Batenburg

**Affiliations:** 1 Computational Imaging, Centrum Wiskunde en Informatica, Science Park 123, 1098 XG Amsterdam, The Netherlands; 2 Faculteit Wiskunde en Informatica, Technical University Eindhoven, Groene Loper 5, 5612 AZ Eindhoven, The Netherlands; 3 Mathematical Institute, Utrecht University, Budapestlaan 6, 3584 CD Utrecht, The Netherlands; 4 Leiden Institute of Advanced Computer Science, Leiden University, Niels Bohrweg 1, 2333 CA Leiden, The Netherlands

**Keywords:** Computational science, Imaging techniques, X-rays

## Abstract

Although X-ray imaging is used routinely in industry for high-throughput product quality control, its capability to detect internal defects has strong limitations. The main challenge stems from the superposition of multiple object features within a single X-ray view. Deep Convolutional neural networks can be trained by annotated datasets of X-ray images to detect foreign objects in real-time. However, this approach depends heavily on the availability of a large amount of data, strongly hampering the viability of industrial use with high variability between batches of products. We present a computationally efficient, CT-based approach for creating artificial single-view X-ray data based on just a few physically CT-scanned objects. By algorithmically modifying the CT-volume, a large variety of training examples is obtained. Our results show that applying the generative model to a single CT-scanned object results in image analysis accuracy that would otherwise be achieved with scans of tens of real-world samples. Our methodology leads to a strong reduction in training data needed, improved coverage of the combinations of base and foreign objects, and extensive generalizability to additional features. Once trained on just a single CT-scanned object, the resulting deep neural network can detect foreign objects in real-time with high accuracy.

## Introduction

X-ray imaging is widely used for non-destructive and high-throughput product quality control of agricultural^1–3^ and manufacturing^4^ products. Because of the unique penetration properties of X-rays, they are well suited for detecting unwanted “foreign objects” that have strong density contrast in a single X-ray view. In this paper, we use the term *foreign object* (FO) in a general manner, referring to the presence of undesirable structures within a certain base product. Examples of such objects include insect infestation in grain^5^, bones in fish^6^ and chicken^7^ fillets, but also structural damages such as fractures in light-alloy casting^8^ and porosity in welds^9^. On a single X-ray view, different object features overlap with each other and make image analysis complicated even for a human expert. While clearly delineated, dense foreign objects could be detected by an observer or a simple algorithm, defects with a complex shape or low attenuation contrast with surrounding materials require more sophisticated approaches.

Deep convolutional neural networks (DCNNs) are the state-of-the-art methods for complex image segmentation and analysis tasks by learning from a sizeable set of high-quality, annotated training images (so-called *supervised learning*^10^). However, while training data for standard computer vision tasks are readily available, high-quality annotated X-ray image data is cumbersome to obtain for industrial quality control applications. Moreover, the combination of changes in the structure of products and foreign objects from one batch to another can render the use of deep learning approaches infeasible, as the amount of data required for retraining the network is prohibitively large.

In this paper, we present a simple yet powerful approach that overcomes these limitations, providing a fast, data-efficient deep-learning strategy for real-time detection of foreign objects in industrial products with high-throughput. Our approach relies on CT-scanning of a few—or even just one—product to create a basic digital product model that can be used for image simulation. By then modifying the CT volume—also including a variety of digital foreign object configurations—and computing artificial X-ray projections of these generated structures, a large training dataset is artificially created that provides an extensive sampling of the combinations of base and foreign objects.

Our experimental results show that although our training data is generated using a simple physics model for X-ray simulation, the model trained on such artificial images can be used effectively on real-world X-ray data, providing accurate and real-time foreign object detection from single-image X-ray views. As a source of real X-ray images, we use datasets containing X-ray projections of pieces of modeling clay with pebble stones inside^11^, and also X-ray CT scans of avocado fruits. We formulate a foreign object detection problem (later referred as FOD) that should be solved with a single X-ray image, and the correct prediction (ground-truth) is defined based on the properties of studied samples. For both case studies, we present two generation strategies that require a different amount of expert knowledge . The basic modification strategy uses a limited number of transformations and reduces the amount of real data acquisition by roughly 50% while preserving the model’s accuracy. We also present a more complex generative model based on a CT scan of just one object that achieves the same accuracy level that would otherwise require tens of samples. The generation of artificial data gives the user full control over sample features in the training set, improving detection accuracy when working with small real-world datasets.

## Related work

X-ray imaging is successfully applied to foreign object detection in a variety of applications. Fish bones could be detected with an accuracy of 99% using conventional image processing and feature extraction^6^, and CNNs can segment bones with 75% per-pixel accuracy^12^. Different stages of pest infestation can be distinguished in soybeans, reaching 86% accuracy of uninfested sample classification^13^. In poultry, X-ray projections are often combined with laser imaging systems to detect bones in fillet samples with more than 95% accuracy^7^. Manually defined features of X-ray images can be used to achieve 85% accuracy of welding defect classification with 5 types of defects^9^. CNNs can provide mean average precision of 92% for localization of casting defects^8^.

Depending on the specific application, the foreign object detection problem can be formulated in different ways. The direct approach is to *detect* whether a foreign object is present in the X-ray image. The outcome could be either binary (yes/no) or from a discrete set of options (multiple FOs that might be present). Additionally, FO detection can be framed as a binary segmentation, where every pixel of the image should be classified as a defect or not. Alternatively, a *semantic segmentation* can be performed by computing the full clusters of pixels containing a single FO.

Several authors have proposed to apply deep learning approaches to FO detection in X-ray images^8,14,15^. Such approaches could achieve high accuracy if a significant amount of annotated training data is provided. The data requirement can be a key obstacle in practical use since they must represent a broad set of possible base objects and foreign objects in different locations. For a segmentation task, the annotation of a large amount of data becomes an additional problem requiring a human expert. The approach in^14^ required 180 pear scans, and 51 volumes were segmented manually. The GDXray dataset^16^ used in^8^ contains 2727 X-ray images of automotive parts and includes annotations of bounding boxes of defects. For the case study in^15^, a dataset of 4725 X-ray images with ground-truth for binary segmentation was used.

In^17^, it was proposed to perform CT scans of objects to generate data for foreign object detection (FOD) in X-ray images. Every scan contains a large number of individual projections that can be used as inputs for FOD. A possibility to perform volume reconstruction and create a 3D model of the sample can be used to generate ground-truth automatically. With this methodology, imaging of thousands of objects can be replaced with tens of CT scans for certain FOD problems, such as fruit inspection. However, reaching a sufficient number of scans might be infeasible if the problem involves multiple FOs. The base objects and all FOs need to be represented by a variety of data, and every CT scan captures a single combination of the base object with a particular FO. Thus, the training data would require all possible pairs that cover a variety of base objects combined with every variation of each FO, leading to a combinatorial explosion.

In many fields, the possibility to create artificial data is considered to overcome the challenge of acquiring large datasets. Monte-Carlo simulations using 3D models of objects of interest were proposed for a variety of manufacturing defect detection problems^4^, luggage inspection^18^, breast X-ray imaging^19^. While this approach could generate realistic data, it requires detailed knowledge of the X-ray imaging setup (source and detector properties) and objects that are supposed to be studied (3D model and material properties). Several studies^12,20^ indicate that realistic synthetic data can be created with simpler models, such as Beer’s law, and use these data to train accurate deep learning models.

## Methods

**Figure 1:**
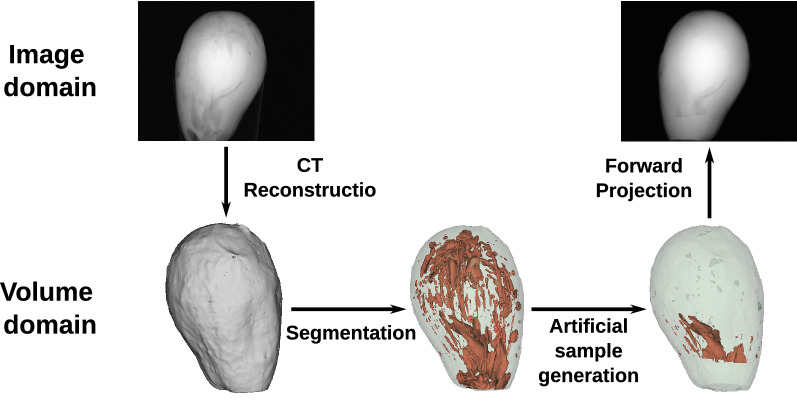
Proposed approach to data generation. All augmentations are performed on volume level and then forward projected to get an X-ray image.

Our approach for creating artificial X-ray images requires at least one CT scan of a product and generates new images by changing the volume of the sample. This methodology is illustrated in Fig. 1. A set of X-ray projections can be converted into a sample volume using CT reconstruction algorithms. The volume is then segmented and deformed to automatically generate a variety of artificial volumes. Finally, X-ray images are computed based on artificial volumes using a forward projector.

In X-ray CT, the object *reconstruction* produces a 3D distribution of the attenuation coefficient inside the inspected volume. Every voxel of the reconstruction corresponds to a small region of the object, and the attenuation coefficient is mainly defined by the properties of the matter there. Furthermore, the reconstruction contains artifacts (e.g. beam hardening, noise, rings) induced by the difference between the model implied by FDK and the actual physical imaging process.

The next step is a *segmentation* of the reconstructed volume. We consider the sample as a combination of a limited number of materials. In a segmented volume, every voxel corresponds to a single material. Both the base object and FO could consist of multiple materials, and on segmentation they can be separated from each other. Additionally, the attenuation coefficient of every material is extracted from the data, by computing an average intensity of reconstructed volume using segmentation as a mask. Thus, any segmented volume can be converted back to the 3D distribution of attenuation using average intensities of materials.

*Artificial sample generation* is performed to create new object volumes similar to the real-world samples. Deep learning models construct a feature space based on the training data, and a successful generation strategy should provide a diverse range of objects to train an accurate model. The main challenge lies in identifying which differences between samples are important and how to make a representative set that covers a variety of possible products that might be encountered by a deep learning model.

**Figure 2:**
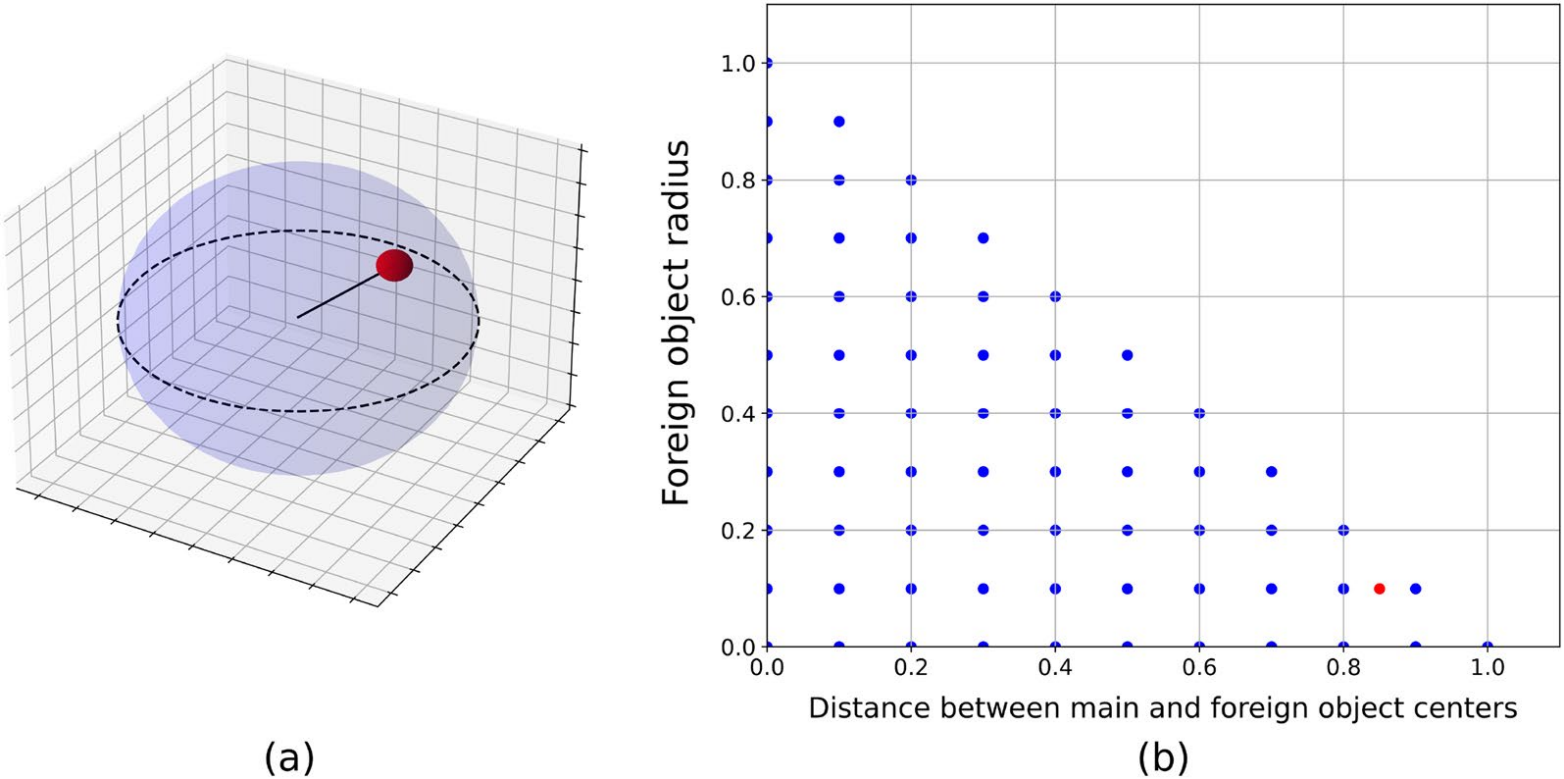
Example of FOD problem where a small ball is located inside a bigger one. Each sample can be described using four parameters, namely radius of the inner ball and three coordinates of the vector connecting centers of the objects. (**a**) One possible sample of this dataset. (**b**) Subspace of configuration space corresponding to the fixed polar and azimuthal angles. The configuration shown on subfigure (**a**) is marked as a red dot in this subspace.

The construction of a representative dataset requires the introduction of prior knowledge about the objects. It provides constraints in the data space and helps to represent every sample as a set of parameters in the configuration space. As a simple example (Fig. 2a), consider an object structure where a unit solid sphere (main object) contains a small ball (foreign object). Due to the symmetries of the objects, every possible sample in this set can be represented as a point in the configuration space with four dimensions, namely the radius of the inner ball and three coordinates of the vector connecting the centers of the objects (subspace of parameters is shown on Fig. 2b). A straightforward generation algorithm for this dataset would evenly sample all possible angles, distances and sizes of the foreign object. Not all parameters are important to produce a variety of images. For example, different polar and azimuthal angles are equivalent to different views of the same object.

In real-world applications, the base object and FOs are usually more complex and cannot be described with a small number of parameters. Some problems, such as detection of cracks during casting or inspection of canned food, have a well-defined main object structure that can be used as a constraint and simplify parameterization. We aim to formulate a more general generation strategy that does not use such constraints. Instead of attempting to map a low-dimensional configuration space and sample objects from it, we propose to apply deformations to the known real volumes. For a particular deformation model with different possible parameters, the output volumes cover a region around the original volume in the data space. We assume that with a variety of deformations we can cover enough data space to train an accurate deep learning model and validate this assumption in the experiments.

Finally, the *forward projection* operator can be used to create artificial projections of the modified volume. The proposed methodology uses a simple approach to forward projection, in which X-ray attenuation follows Beer’s law, and the image is calculated by computing line integrals. This method does not take into account many experimental effects, such as a polychromatic spectrum of the tube and detector noise. We additionally apply post-processing to the noiseless projections in order to make them more similar to real data. Mixed Poisson–Gaussian noise is applied to every pixel according to

1 $$\begin{aligned} I_{noisy} \sim \frac{1}{\lambda } {\mathscr {P}} (\lambda I_0) + {\mathscr {N}}(0, \sigma ^2), \end{aligned}$$

where $I_0$ is a noiseless photon count, $\lambda$ and $\sigma$ are noise parameters. The values of these parameters can be found by performing a calibration of the detector^21^. As a result, the forward projector can be computed significantly faster than a Monte–Carlo simulation, and the data have a noise distribution roughly imitating the real one.

## Results

### Deep learning model: training, validation and accuracy evaluation

As an example of deep learning model, we train DCNNs with EfficientNet B4^22^ architecture and cross-entropy loss to perform classification of X-ray images. The network is trained with different numbers of training images $N_{train}$ to evaluate how the size of the training set affects the network accuracy *A*. The accuracy is determined by applying the model to the same set of $N_{test}$ images and comparing true classes ${y_i}$ and predicted classes $\hat{y_i}$. The score *A* is computed according to the formula

2 $$\begin{aligned} A = \frac{1}{N_{test}} \sum _{i=0}^{N_{test}-1} \mathbbm {1}(\hat{y_i} = y_i), \end{aligned}$$

where $\mathbbm {1}(x)$ is the indicator function. The test set contains equal number of images for every class to ensure that the accuracy score is not biased towards certain classes.

In both case studies, there is a a collection of experimentally acquired CT scans split between training ($S_{train}$ samples) and test ($S_{test}$ samples). The test set always consists of real data, *p* projections per sample, resulting in $N_{test} = p S_{test}$. When the network is trained on real data, the training set is constructed similarly: $N_{train} = p S_{train}$. To evaluate the data generation performance, the network is trained only on artificial data, and the same number of projections is created with fewer volumes: $N_{train} > p S_{train}$. The goal of the data generation method is to achieve similar *A* with fewer $S_{train}$.

Training of the network stops based on the validation score. The training data are split in advance between training and validation subsets, approximately 15% of images is used for validation. The performance of the trained neural network might significantly depend on random factors, such as initialization and nondeterministic algorithms. As a result, the value of *A* changes if the same network is trained multiple times. To characterize the uncertainty $\Delta A$, every model is trained four times with different random seeds and the same training data.

### Data

The CT-based approach for creating artificial X-ray images is illustrated on two FOD problems. The first case study is based on the dataset^11,17^ that was acquired at the Computational Imaging group of Centrum Wiskunde en Informatica (CWI) in Amsterdam, The Netherlands. These data were collected as an example of X-Ray FOD and contain images of modeling clay with pebble stones. We use these data as a model problem for FOD with a piece of clay being a homogeneous main object and stone as a foreign object. Even though this problem does not have an inherent industrial value, it poses challenges similar to industrial inspection tasks, such as detection of bones in poultry or glass in peat. Firstly, a piece of clay was remolded for every scan, so the dataset contains a variety of main objects with unpredictable shape. Secondly, despite a significant contrast, a pebble stone can still be not detected on a single projection due to unknown shape of the sample and small size of the stone.

**Figure 3:**
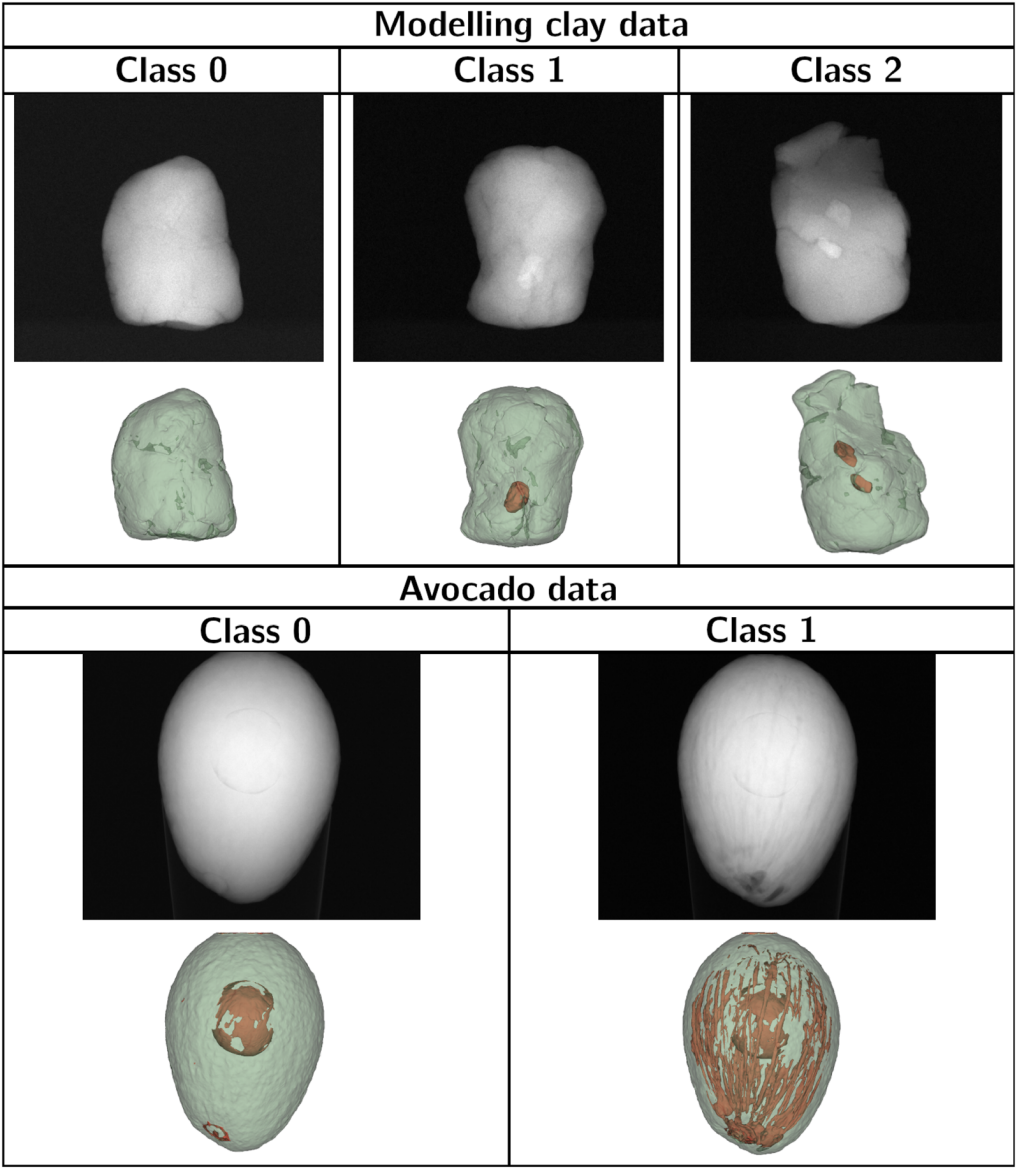
Examples of objects from differenct classes for two case studies. The first row corresponds to input X-ray images, and the second row shows visualization of the volume based on the segmentation (base object is colored green, foreign object—red).

The training and test set contain $S_{train}=30$ and $S_{test}=30$ samples from the original collection with zero, one, and two stones. For a classification task, the number of foreign objects in the sample is chosen as a class (examples of all classes are shown on Fig. 3). We have additionally acquired CT scans of 10 samples without pebble stones since the original dataset only had 11 such scans. The experimental settings of these additional scans are similar to the settings in the original collection. For every sample, $p=72$ projections are used as X-ray images for FOD. Furthermore, the original data are downsampled with a factor of 2, for an effective pixel size of 300 $\upmu m$ and voxel size of $190\, \upmu$m.

To acquire the second dataset, we performed X-ray scans of twenty five avocado fruits at the FleX-ray laboratory^23^ of CWI in Amsterdam, The Netherlands. The measurements were performed with the voltage of 90 kV, power of 45 W, exposure time of 300 ms per projection, and magnification factor of 1.3. The original X-ray image size was $1912 px \times 1520 px$ with a pixel size of 75 $\upmu m$, 1440 images were acquired for every sample. The fruits were stored without refrigeration for 2 weeks and scanned throughout this period. We observed that over time the amount of air inside avocado was increasing. Thus, a freshly harvested fruit can be distinguished from a ripe one based on the presence of air. Speed of this process significantly varies among different samples due to inherent biological variability. To formulate a FOD problem, we consider presence of air pockets inside avocado as a foreign object.

The experimental dataset contains $S_{train}=36$ and $S_{test}=20$ objects (most fruits have multiple scans corresponding to different days), $p=72$ projections per object. For every sample, a class is assigned based on the fraction of air volume with respect to the whole fruit. We have chosen an arbitrary threshold of 1% air to separate two classes of objects (examples of both classes are shown on Fig. 3). For faster deep learning model training, images and reconstructions were downsampled with a factor of 4, leading to the effective pixel size of 300 $\upmu m$ and voxel size of $230 \upmu m$.

### Sample generation

Data generation reduces the amount of manual data acquisition by constructing a representative training set consisting of artificial X-ray images. This approach could only work if the artificial and real data are similar enough, so the trained model can be applied to real images. To evaluate similarity, we train the same DCNN on real images and artificial images that are supposed to be identical to real. More precisely, we perform the segmentation and forward projection without any changes of the volume. First, this procedure checks if the segmentation is accurate enough to preserve contrast and morphology of object features. Second, we evaluate how much missing physical effects influence the training process of DCNN. Furthermore, this comparison verifies if the addition of noise could mitigate shortcomings of the simple forward projection operator.

In the presented case studies, formulating a deformation model is a challenging task since neither base nor foreign object have a fixed shape or blueprint. Thus, it is not trivial to check algorithmically if the deformed object is still an object of the same type that can be encountered by the inspection system (e.g. an avocado fruit after deformation is still avocado). The first deformation model we propose to use is an affine transformation that includes resizing, rotation, shear and translations. By applying it to the foreign object, it is possible to change its placement inside the base object and generate a variety of volumes using a single scan as a blueprint. Furthermore, resizing and shear could slightly change the shape of the object while preserving morphological features since the size of agricultural products often varies in a certain range. However, such reasoning might break if the transformation matrix is sufficiently different from identity matrix (e.g. a deformed object is orders of magnitude smaller than the original one), so a manual inspection of deformed objects is necessary to determine a range of parameters. It is possible that this range of parameters is too small to produce visibly different images rending affine transformation unsuitable for some FOD problems. For example, in avocado case study, air pockets might form around specific regions of the fruit, and translations are not always applicable. To reflect this case, a region removal method is used as a non-linear deformation model. This algorithm splits any object into regions by randomly assigning a number of point in the object to different clusters and then performing a nearest neighbor search for all other points. Then a certain number of these clusters is removed and the rest is kept. The region removal method is used to change the shape of the object considerably while preserving its position.

For both case studies, we present two possible generation strategies that require different amount of real data and expert knowledge to define deformation models. The first one (later referred as basic modification) takes multiple samples of the same class and generates images for other classes by applying deformation models to the foreign object. In the modelling clay case, the algorithm starts with a segmentation of the sample containing two stones (Class 2) and generates two artificial samples: one for Class 1 by removing one of the stones and one for Class 0 by removing both. Pieces of stone are removed by replacing stone material on segmentation with the modelling clay material. Thus, the number of training images $N_{train}$ is $3pS_{train}$ since the samples of only one class are used by the generation method. For avocado FOD problem, the algorithm takes a volume of Class 1 avocado and applies region removal to reduce the amount of air. As a result, every real-world sample is used to create two avocado volumes of both classes, and $N_{train} = 2pS_{train}$. It is crucial to remove only a fraction of air. Otherwise, the network wouldn’t see examples of Class 0 objects with air and misclassify images in the test set.

**Figure 4:**
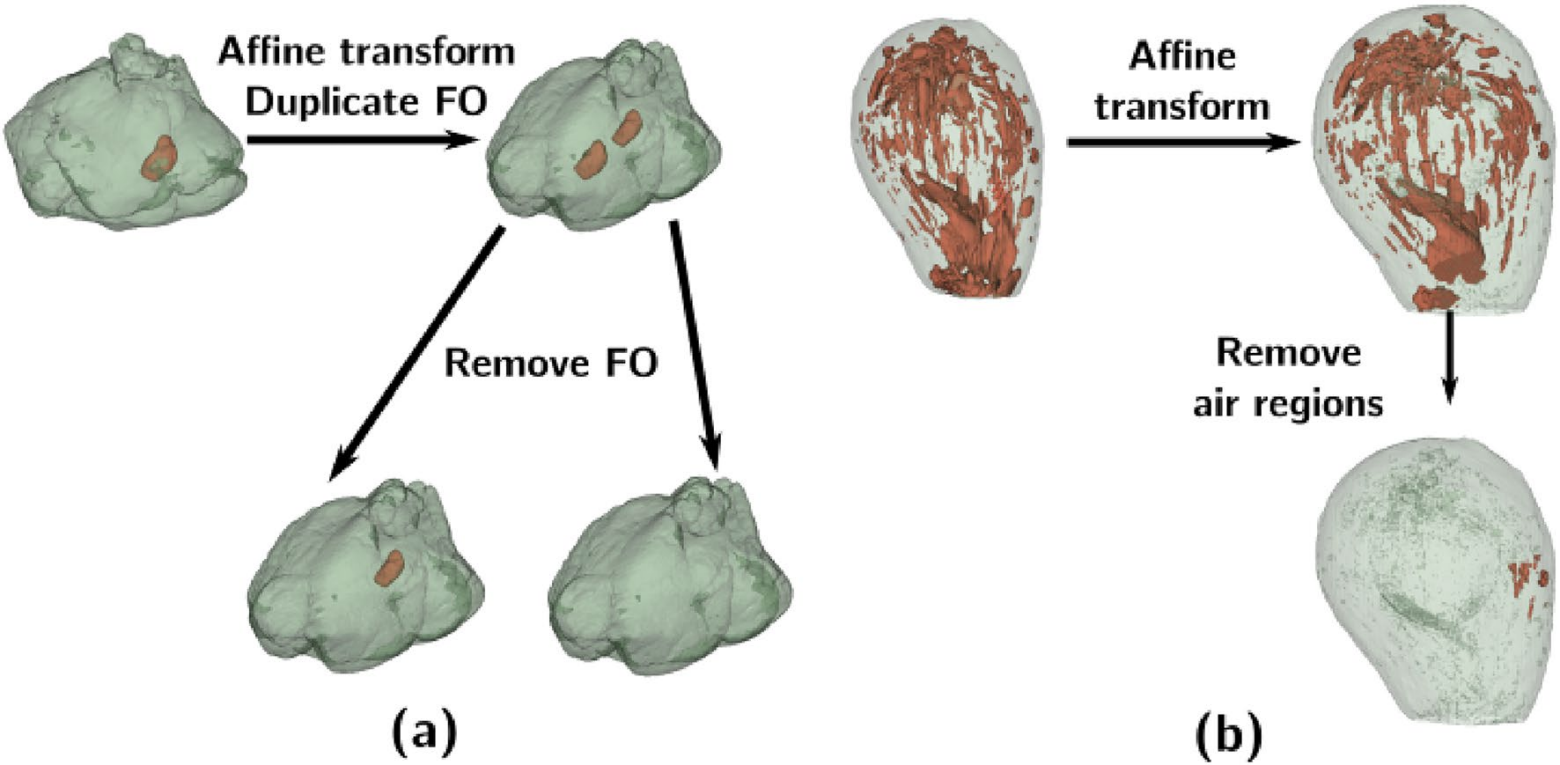
Artificial sample generation based on one real-world object: (**a**) for modelling clay; (**b**) for avocado.

The second, more data-efficient strategy applies deformation models to a single real-world object to generate as many different artificial volumes as possible. The first step is to apply affine transformation (a mix of resizing and shear) to the real volume. Parameter range is chosen by a human expert to ensure that a deformed volume is not identical to the original one, but still looks like a real sample. Thus, scaling coefficients are in a neighborhood of 1, and shear coefficients are around 0. The exact limits used for our implementation can be found in the code repository (referenced in *Code and Data availability*). In the modelling clay case, the original volume is chosen from Class 1. Furhermore, in addition to the affine transformation of the whole object, the pebble stone is duplicated using a second affine transformation. This FO tranformation includes not only resize and shear, but also translation and rotation to create a Class 2 sample with two pebble stones as shown on Fig. 4a. This is an arbitrary choice that highlights that a real sample with two FOs is not required to generate artificial samples with two FOs. Then, as in basic modification, the stone objects are removed from this artificial volume to create Class 0 and 1 samples. For avocado case (Fig. 4b), an affine transformation is applied to the Class 1 sample, and Class 0 objects are generated by performing region removal. In both case studies, the number of generated images $N_{train}$ is $pGS_{train}$, where *G* is the number of artificial volumes generated for every real scan, and $S_{train} = 1$.

### Software implementation

ASTRA Toolbox^24^ is used to perform reconstruction of the sample volumes based on the X-ray CT scans using FDK algorithm^25^. The segmentation utilizes image processing algorithms (Otsu, convex hull, morphological operations) implemented in the scikit-image package^26^. Deformation models apply affine transformation implemented in the SciPy package^27^, and the region removal algorithm was implemented by us. Forward-projection uses the operator from ASTRA Toolbox.

### Accuracy of modeling clay classification

**Figure 5:**
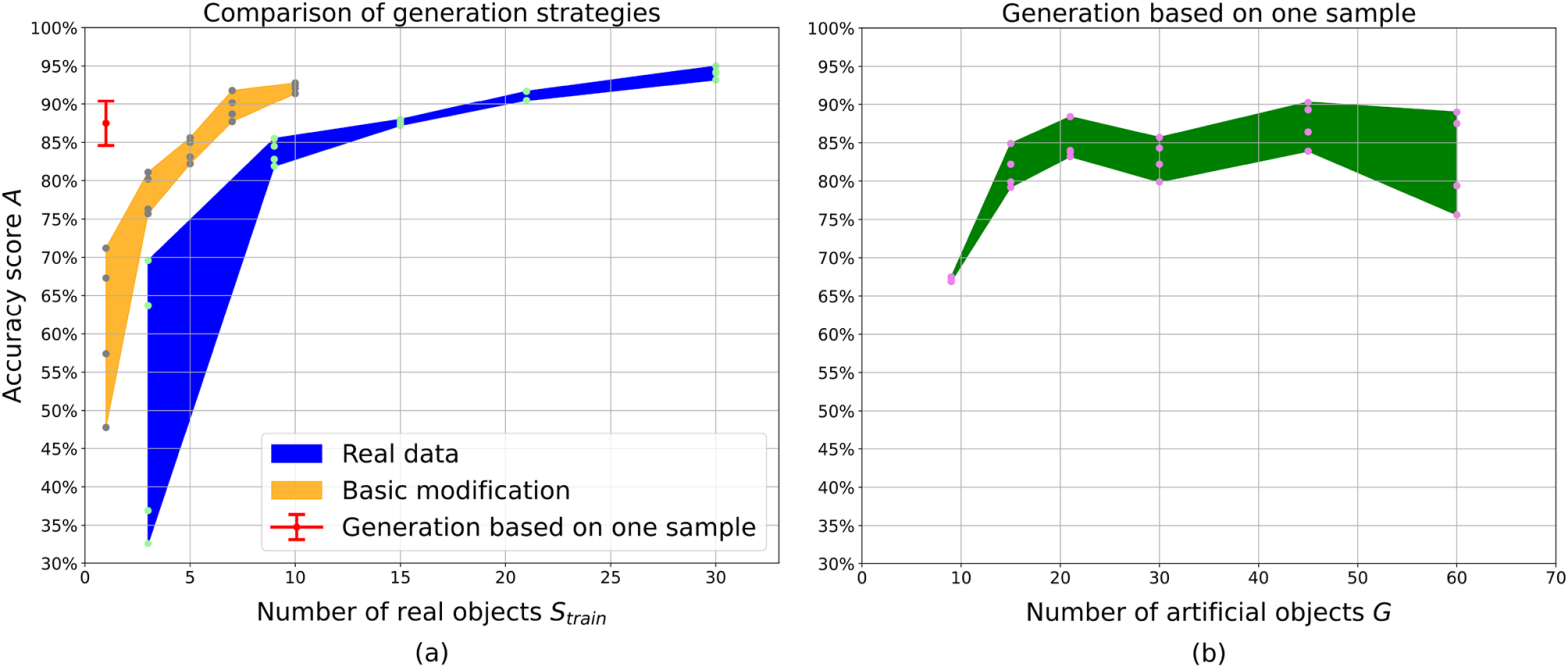
Dependency of modeling clay classification accuracy *A* on the amount of data provided for training. (**a**) Comparison between training on real data and generated images with a different number of real samples $S_{train}$. (**b**) Training on the artificial images generated based on one experimentally scanned sample.

For modeling clay FOD, the DCNN performance for different amounts of real data used for training is drawn as a blue region on Fig. 5a, green dots correspond to the accuracy scores *A* of the individual instances of the same network. Training images based on $S_{train}>20$ CT scans are enough to achieve $A= 90\%$. Quality of the artificial data was tested on a subset of $S_{train} = 21$ real-world samples. When the model is trained on generated images corresponding to the real volumes, the average *A* stays on the same level of $\approx 90\%$ as with real data. The values of *A* for noiseless and noisy artificial projections are shown in Table 1. Mixed noise is applied according to Eq. (1) with the values of noise parameters $\lambda = 1.17$ and $\sigma =20$. Simulation of noise reduces $\Delta A$, but does not improve the average performance.

The accuracy score of networks trained on generated data is shown as orange region (basic modification) and a red point (generation from one) on Fig. 5a. For basic modification, the values of *A* approximately correspond to accuracy score achieved with twice as many real samples. Thus, this generation strategy reduces the amount of data acquisition by half and achieves the same predictive strength.

For the generation from one sample ($S_{train}$ = 1), the value *A* mainly depends on the number of artificial volumes *G* generated from this sample (Fig. 5b). For $G < 20$ the resulting performance rises and then saturates. The best value of $A = 87\% \pm 3\%$ is achieved with $G = 45$ and corresponds to the performance that could otherwise be achieved with $S_{train} = 15$ real objects. The important difference is that $\Delta A$ does not decrease when the number of generated objects *G* rises as opposed to real data. Furthermore, with $G > 60$ the mean value of *A* is getting worse due to a higher $\Delta A$.

**Table 1:** Comparison of model accuracy for different types of training data: real images, artificial noiseless images, and artificial images with noise added as a post-processing filter.

Training data, $S_{train} = 21$	Accuracy score *A*
Real data	$91.1\% \pm 0.7\%$
Noiseless artificial	$91.8\% \pm 1.3\%$
Noisy artificial	$91.8\% \pm 0.4\%$

### Accuracy of avocado classification

**Figure 6:**
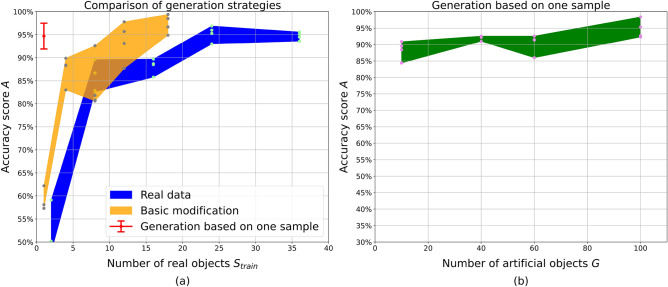
Dependency of avocado classification accuracy *A* on the amount of data provided for training. (**a**) Comparison between training on real data and generated images with a different number of real samples $S_{train}$. (**b**) Training on the artificial images generated based on one experimentally scanned sample.

According to the experiments with real data (Fig. 6a), scans of $S_{train} = 24$ fruits are enough to achieve binary classification accuracy $A = 95\%$. The standard deviation $\Delta A$ decreases to 2% and stays at that level when more data are used for training. The subset of $S_{train} = 24$ objects was also used to check the quality of the forward projection operator. As shown in Table 2, the network accuracy significantly drops if the model is trained on noiseless data. However, the addition of mixed Poisson–Gaussian noise improves the performance of the model. The difference in accuracy between models trained on real and noisy artificial images lies in one standard deviation range.

The accuracy score of networks trained on generated data is shown as an orange region (basic modification) and a red point (generation from one) on Fig. 6a. Networks trained on images generated with the basic modification strategy reach the best real-data performance $A \approx 95\%$ with $S_{train} = 12$ instead of 24 real objects. Furthermore, this method outperforms training on real data achieving $A = 97\% \pm 2\%$ with all available Class 1 samples. Thus, a 50% reduction in the amount of data acquisition $S_{train}$ is also possible in the avocado case without loss of performance.

The generation from one sample strategy leads to a model accuracy $A = 95\% \pm 3\$$ with $S_{train}=1$. The value of *A* slightly depends on the number of generated volumes *G* (Fig. 6b), but the standard deviation $\Delta A$ stays at 3% even for a large number of $G > 50$. In the avocado case, the exact values of *A* significantly depend on the parameters used for air region removal. A variety of Class 0 samples can be generated from a Class 1 sample since air clusters to be removed can be chosen in different ways. For this reason, the experiments with data generation were repeated multiple times with different random seeds and distributions for the amount of air to be removed from the sample. In case of generation from one, the experiment was also performed with a different original object. With a small number of $S_{train}$ for basic modification and small *G*, the values of *A* could drop significantly with respect to the reported $\Delta A$. However, by generating $G = 100$ artificial volumes it was possible to reach the performance of $A > 92\%$ with different random distributions and original real-world volumes.

**Table 2:** Comparison of model accuracy for different types of training data: real images, artificial noiseless images, and artificial images with noise added as a post-processing filter.

Training data, $S_{train} = 21$	Accuracy score *A*
Real data	$95.3\% \pm 1.7\%$
Noiseless artificial	$90.5\% \pm 4.4\%$
Noisy artificial	$94.5\% \pm 1.5\%$

## Discussion

The results show that for two case studies with real data, accurate classification models can be trained on artificial X-ray images based on a small amount of data acquisition. For modelling clay case, one experimentally scanned object provides enough information for the generation method to achieve accuracy corresponding to the training on 15 real samples. Despite using only one real-world avocado, the model trained on generated fruits reaches the performance that would otherwise require more than 20 objects. Such a significant reduction in the amount of data acquisition requires a change in data acquisition approach and poses new application-specific challenges related to data generation. To effectively apply this methodology, the user has to guide the acquisition of CT data, formulate deformation models, and adjust the forward projection operator.

Despite FOD problem being formulated for a single X-ray projection, this methodology specifically requires CT scans of samples that provide enough data to reconstruct volumes. Although in the case studies a laboratory setup was used for both tasks, in the industrial case they should be performed with two separate systems. A detector on a factory floor is used to take low-resolution images with low exposure for a fast FOD, and a CT scanner acquires a large number of projections from different angles with high exposure and resolution. Even though a CT scan takes more time than a single projection, the data generation approach requires fewer real-world samples with a proper choice of deformation models.

Access to 3D models of base and foreign objects gives user a direct control over all possible combinations that can be encountered by a quality control system. Deformation models applied to the segmented volume can be used to put foreign objects in different locations of the base object, and transformations of the base object can change features visible on a single X-ray view. In the manual data acquisitions, this variability would require the user to provide many different samples representing different combinations of the base object and FO. For FOs of biological nature (air gaps, infestation, chemical changes in tissues), it is not possible to construct an arbitrary sample in real life, but it can be generated algorithmically by providing necessary models. The main limitation of the sample generation approach lies in the application-specific expert knowledge to make sure that a variety of possible samples is represented. Additionally, artificial volumes might be impossible to exist in reality, due to biological or physical constraints, and such samples could mislead the model and decrease its predictive ability.

Artificial sample generation can be utilized to reuse the existing data and extend problem formulation. Possibility to deform sample volumes can be used to adapt the existing deep learning model to another size of FOs or a different shape of the base objects. New types of FOs can be scanned separately and injected in the existing models of the base objects. Data generation strategies with many real-world samples, such as basic modification, can be used to fix class imbalance in the manually acquired datasets.

The data generation approach relies on the assumption that artificial X-ray projections can be similar to real projections. Depending on the application, the user has to adjust a forward projection operator. In the presented case studies, the operator did not include many physical effects that can be simulated with Monte-Carlo methods. Nevertheless, the results show that a more accurate operator would not significantly improve the accuracy of the deep learning model. Even though we did not compare our approach with other simulation methods, we evaluated the performance based on real data, and a perfect operator would replicate real images. Good enough performance of the simple operator can be explained by a low resolution of the inspected images and structure of the deep learning models that might ignore or blur small details.

A significant challenge of this methodology is its sensitivity to the application-specific decisions made by the user. Some implementation details might significantly change the generated data, and this effect can only be evaluated by training a deep learning model. Such behavior was observed for artificial avocados where the amount of air in the generated volumes could significantly change the resulting accuracy of the model. However, a similar problem can be highlighted for conventional training on real data where the performance is implicitly controlled by the acquired images. Picking a representative set of real-world samples is also a challenge, especially if the different batches of products have different FO patterns. The data generation approach provides a more direct way to control coverage of the training data and a faster way to expand the existing dataset.

## Conclusion

Efficient application of deep learning methods to industrial quality control requires large annotated datasets that are rarely available. We present a CT-based method for creating artificial X-ray images that can be used to train DCNNs instead of real data. This methodology requires a small number of CT scans, significantly reducing the amount of necessary data acquisition. Deformation models applied to the segmented sample volumes generate a variety of possible combinations of the base and foreign objects and create a representative dataset. The sample generation strategy can be adjusted based on the amount of available data and a-priori knowledge about the problem and makes the approach flexible to new features in the data and extensions of the problem formulation. The results show that even a single CT scan can train a model capable of accurate foreign object detection in real-time. By changing the approach to data acquisition, this methodology simplifies the use of deep learning methods for industrial quality control tasks.

## Data Availability

Compressed versions of both datasets are available on Zenodo: https://doi.org/10.5281/zenodo.6901633. The data include binned pre-processed projections and segmented volumes.

## Code availability

The code used for artificial X-ray image generation can be found on Github: https://github.com/vandriiashen/flexsim. The code for deep learning classifier training and application can be found in another repository: https://github.com/vandriiashen/aug_accuracy.

## References

[1] Haff RP, Toyofuku N (2008). X-ray detection of defects and contaminants in the food industry. Sens. Instrum. Food Qual. Saf..

[2] Mathanker SK, Weckler PR, Bowser TJ (2013). X-ray applications in food and agriculture: A review. Trans. ASABE.

[3] Kotwaliwale N (2014). X-ray imaging methods for internal quality evaluation of agricultural produce. J. Food Sci. Technol..

[4] Mery D (2015). Computer Vision for X-ray Testing.

[5] Divyanth L, Chelladurai V, Loganathan M, Jayas DS, Soni P (2022). Identification of green gram (*Vigna radiata*) grains infested by *Callosobruchus maculatus* through X-ray imaging and gan-based image augmentation. J. Biosyst. Eng..

[6] Mery D (2011). Automated fish bone detection using X-ray imaging. J. Food Eng..

[7] Chen X, Jing H, Tao Y, Cheng X (2005). Pattern classification for boneless poultry inspection using combined x-ray/laser 3d imaging. Optical Sensors and Sensing Systems for Natural Resources and Food Safety and Quality.

[8] Ferguson, M., Ak, R., Lee, Y.-T. T. & Law, K. H. Automatic localization of casting defects with convolutional neural networks. In *2017 IEEE International Conference on Big Data (Big Data)*, 1726–1735 (IEEE, 2017).

[9] Duan F (2019). Automatic welding defect detection of X-ray images by using cascade adaboost with penalty term. IEEE Access.

[10] Rawat W, Wang Z (2017). Deep convolutional neural networks for image classification: A comprehensive review. Neural Comput..

[11] Zeegers, M. T. A collection of 131 ct datasets of pieces of modeling clay containing stones—part 1 of 5. *Zenodo*. 10.5281/zenodo.5866228 (2022).

[12] Urazoe K (2021). Automated fish bone detection in X-ray images with convolutional neural network and synthetic image generation. IEEJ Trans. Electr. Electron. Eng..

[13] Chelladurai V, Karuppiah K, Jayas D, Fields P, White N (2014). Detection of *Callosobruchus maculatus* (f.) infestation in soybean using soft X-ray and NIR hyperspectral imaging techniques. J. Stored Prod. Res..

[14] Van De Looverbosch T, Raeymaekers E, Verboven P, Sijbers J, Nicolai B (2021). Non-destructive internal disorder detection of conference pears by semantic segmentation of X-ray ct scans using deep learning. Expert Syst. Appl..

[15] Lee D-H, Kim E-S, Cho J-S, Ryu J-H, Min B-S (2022). A two-stage automatic labeling method for detecting abnormal food items in X-ray images. J. Food Meas. Charact..

[16] Mery D (2015). Gdxray: The database of X-ray images for nondestructive testing. J. Nondestr. Eval..

[17] Zeegers, M. T. *et al.* A tomographic workflow to enable deep learning for x-ray based foreign object detection. arXiv:2201.12184 (arXiv preprint) (2022).

[18] Gong Q (2018). Rapid simulation of X-ray transmission imaging for baggage inspection via gpu-based ray-tracing. Nucl. Instrum. Methods Phys. Res. Sect. B.

[19] di Franco F (2020). Geant4 monte carlo simulations for virtual clinical trials in breast X-ray imaging: Proof of concept. Phys. Med..

[20] Van De Looverbosch T (2022). Inline nondestructive internal disorder detection in pear fruit using explainable deep anomaly detection on X-ray images. Comput. Electron. Agric..

[21] Konstantinidis AC, Szafraniec MB, Speller RD, Olivo A (2012). The dexela 2923 cmos X-ray detector: A flat panel detector based on cmos active pixel sensors for medical imaging applications. Nucl. Instrum. Methods Phys. Res. Sect. A.

[22] Tan, M. & Le, Q. Efficientnet: Rethinking model scaling for convolutional neural networks. In *International Conference on Machine Learning*, 6105–6114 (PMLR, 2019).

[23] Coban SB, Lucka F, Palenstijn WJ, Van Loo D, Batenburg KJ (2020). Explorative imaging and its implementation at the flex-ray laboratory. J. Imaging.

[24] Van Aarle W (2016). Fast and flexible x-ray tomography using the astra toolbox. Opt. Express.

[25] Feldkamp LA, Davis LC, Kress JW (1984). Practical cone-beam algorithm. Josa A.

[26] Van der Walt S (2014). scikit-image: Image processing in python. PeerJ.

[27] Virtanen P (2020). Fundamental algorithms for scientific computing in Python SciPy 1.0.. Nat. Methods.

